# Real-Time Mobile Monitoring of Drinking Episodes in Young Adult Heavy Drinkers: Development and Comparative Survey Study

**DOI:** 10.2196/13765

**Published:** 2019-11-20

**Authors:** Daniel J Fridberg, James Faria, Dingcai Cao, Andrea C King

**Affiliations:** 1 Department of Psychiatry and Behavioral Neuroscience The University of Chicago Chicago, IL United States; 2 Department of Ophthalmology The University of Illinois at Chicago Chicago, IL United States

**Keywords:** ecological momentary assessment, young adults, binge drinking

## Abstract

**Background:**

Binge drinking, defined as consuming five or more standard alcoholic drinks for men (four for women) within a 2-hour period, is common among young adults and is associated with significant alcohol-related morbidity and mortality. To date, most research on this problem in young adults has relied upon retrospective questionnaires or costly laboratory-based procedures. Smartphone-based ecological momentary assessment (EMA) may address these limitations by allowing researchers to measure alcohol use and related consequences in real time and in drinkers’ natural environments. To date, however, relatively less research has systematically examined the utility of this approach in a sample of young adults targeting real-world heavy drinking episodes specifically.

**Objective:**

This study aimed to evaluate the feasibility, acceptability, and safety of a smartphone-based EMA method targeting binge drinking and related outcomes in heavy drinking young adults during real-world drinking occasions.

**Methods:**

Young adult binge drinkers in the smartphone group (N=83; mean 25.4 (SD 2.6) years; 58% (48/83) male; bingeing on 23.2% (6.5/28) days in the past month) completed baseline measures of alcohol use and drinking-related consequences, followed by up to two smartphone-based EMA sessions of typical drinking behavior and related outcomes in their natural environments. They also completed next-day and two-week follow-up surveys further assessing alcohol use and related consequences during the EMA sessions and two weeks after study participation, respectively. A separate demographic- and drinking-matched safety comparison group (N=25) completed the baseline and two-week follow-up surveys but did not complete EMA of real-world drinking behavior.

**Results:**

Most participants (71%, 59/83) in the smartphone group engaged in binge drinking during at least one 3-hour EMA session, consuming 7.3 (SD 3.0) standard alcoholic drinks. They completed 87.2% (507/581) system-initiated EMA prompts during the real-world drinking episode, supporting the feasibility of this approach. The procedure was acceptable, as evidenced by high participant ratings for overall satisfaction with the EMA software and study procedures and low ratings for intrusiveness of the mobile surveys. Regarding safety, participants endorsed few drinking-related consequences during or after the real-world drinking episode, with no adverse or serious adverse events reported. There were no differences between the groups in terms of changes in drinking behavior or consequences from baseline to two-week follow-up.

**Conclusions:**

This study provided preliminary support for the feasibility, acceptability, and safety of a smartphone-based EMA of real-time alcohol use and related outcomes in young adult heavy drinkers. The results suggest that young adults can use smartphones to safely monitor drinking even during very heavy drinking episodes. Smartphone-based EMA has strong potential to inform future research on the epidemiology of and intervention for alcohol use disorder by providing researchers with an efficient and inexpensive way to capture large amounts of data on real-world drinking behavior and consequences.

## Introduction

### Background

Binge drinking, also known as heavy episodic drinking, is a significant public health problem in the United States and accounts for more than half of the 88,000 alcohol-related deaths in the United States each year [1,2]. Approximately 25% of young adults aged 18 to 34 years report past-month binge drinking, defined as consuming 5 or more standard alcoholic drinks for men (4 for women) within a 2-hour period [3], which is the highest prevalence rate across the life span [1]. These individuals are at significant risk for developing alcohol use disorder (AUD) and morbidity and mortality related to excessive alcohol consumption [4]. Thus, improving our knowledge of the factors that maintain binge drinking behavior in young adults is critical to developing new interventions for AUD. To date, most research on experiences of young adults during binge drinking has either used retrospective surveys (eg, [5]) or laboratory-based alcohol challenge paradigms (eg, [6-8]); however, these approaches are limited by recall bias and unclear ecological validity, respectively. One potential solution to these limitations is the use of ecological momentary assessment (EMA), which allows researchers to use technology such as mobile phones to measure participants’ real-world behaviors in real time and in their natural environments [9]. In this study, we report on the feasibility, acceptability, and safety of smartphone-based EMA targeting binge drinking and related outcomes in young adult heavy drinkers’ natural environments.

Prior research has used EMA to measure real-world light-to-moderate alcohol use (ie, 2-4 standard drinks consumed on average) with good compliance across studies (ie, 78-90% of survey prompts completed) [10-15]. EMA methodologies in drinkers have varied from random prompts of mood and behaviors throughout the day to prompts specifically targeting drinking episodes. Most of this work has been conducted in general samples of young adult drinkers and not specifically in hazardous drinkers during binge drinking episodes specifically, although prior studies have captured EMA data on heavy drinkers and binge episodes through recruitment of broader samples of young adult drinkers generally (refer to the studies by, eg, Kuntsche and Labhart, Piasecki et al, and Groefsema et al [12,14,16]). Although EMA methods are acceptable in young social drinkers [13], with little [13] or no effect [11] on subsequent drinking behavior (ie, reactivity to the assessment method), it is unclear whether such acute monitoring of real-world drinking will also be feasible and safe in young adult chronic heavy drinkers who regularly drink to intoxication. To advance the field and increase the clinical relevance of EMA methods of monitoring drinking in hazardous users, additional work is needed on the acceptability of the procedure and reactivity to EMA in young adult heavy drinkers and others at risk for AUD [17].

Given our current era of rapid advances in mobile technology and changing user preferences, the modality of alcohol monitoring is important. Most prior EMA studies of drinking have employed either palmtop computers [14] or older cellular phone technology such as texting (eg, [12]). However, recent advancements in smartphone technology and the popularity of these devices (94% of young adults aged 18-29 years own a smartphone; [18]) have rendered these older methods obsolete and ushered in a new era for user-friendly, real-time monitoring of participants’ real-world experiences. To our knowledge, however, no previous studies have focused specifically on the ability of smartphone-based EMA to assess real-time drinking behavior and related outcomes in young adult heavy drinkers during binge drinking episodes. Although modern smartphones have several advantages over prior EMA technology in terms of ease of use and familiarity, they also offer numerous distractions competing for users’ attention. As such, it is possible that they may not be a useful platform for assessing alcohol use and other outcomes during a heavy drinking episode.

### Objectives

Therefore, the purpose of this study was to evaluate the feasibility, acceptability, and safety of a real-time smartphone-based EMA method to obtain self-reported alcohol use and related outcomes (drinking context and subjective alcohol responses) during real-world drinking events in young adult heavy drinkers. We targeted the EMA procedure in this study toward binge drinking episodes specifically because previous work in this field has focused mainly on capturing drinking behavior generally over a period, for example, several days or weeks [14,16,19], rather than binge drinking outcomes per se, and because more data are needed to establish the acceptability, feasibility, and safety of EMA of binge drinking episodes specifically. We hypothesized that the method would be feasible, as evidenced by good compliance (ie, ≥80% response rate of survey prompts), and acceptable, as evidenced by high reported satisfaction with study participation and low perceived intrusiveness of the mobile assessments. We also evaluated the safety of the mobile method by examining participants’ drinking behaviors and related consequences before and several weeks after completing the assessments relative to a comparison group who did not undergo monitoring of binge drinking.

## Methods

### Design

The Mobile Alcohol Response Study was conducted between April 2017 and June 2018 and employed a within-subject design with each participant undergoing 1 or 2 separate real-time smartphone assessments of typical drinking episodes in their natural environment. As stated above, a separate safety comparison sample completed baseline and follow-up surveys of drinking behavior and related outcomes but did not undergo real-time mobile monitoring. As part of a larger study, participants also completed a laboratory session with alcohol administration to validate the smartphone-based EMA method; those data will be reported elsewhere. All study procedures were approved by the University of Chicago Institutional Review Board.

### Participants

Candidates were recruited from Web-based advertisements and word-of-mouth referrals. Inclusion criteria were age 21 to 29 years, generally healthy young adults with weekly heavy alcohol consumption (≥5 drinks in 1-4 weekly occasions for men and ≥4 drinks for women; [3]) for at least the past year, and consumption of ≥14 drinks per week for men or ≥7 drinks per week for women, similar to previous studies by our group [6,20-22]. Candidates meeting the basic study criteria from telephone screening were invited to an in-person visit to confirm their eligibility for the study. They were instructed to abstain from alcohol and recreational drugs for 24 hours before screening, and breath alcohol concentration (BrAC) and urine drug toxicology tests (cocaine, amphetamines, methamphetamines, opioids, and benzodiazepines) corroborated recent abstinence from these substances, with the exception of 1 candidate who tested positive for benzodiazepine use and was excluded from further participation. As approximately one-third of young adult binge drinkers report using cannabis [23], current cannabis use was not an exclusion criterion, as long as the reported frequency did not exceed 3 times per week, the candidate agreed to refrain from using it during the real-world mobile drinking monitoring sessions, and they did not meet Diagnostic and Statistical Manual of Mental Disorders, Fifth Edition (DSM-5) criteria for cannabis use disorder.

The screening visit included informed consent, an explanation of study procedures, surveys, and interviews conducted by a trained research assistant*.* The surveys included the Alcohol Use Disorder Identification Test [24], the Beck Depression Inventory [25] and Spielberger Trait Anxiety Inventory [26] to assess current symptoms of depression and anxiety, and a demographic and health history questionnaire. Interviews included the Timeline Followback (TLFB) calendar [27] for past-month alcohol drinking, the Alcohol Quantity-Frequency Interview [28] for typical and maximum drinking over the past 6 months, and the Structured Clinical Interview for the DSM-5, Research Version [29]. Candidates were excluded if they met criteria for severe AUD or a major psychiatric disorder. Identical recruitment methods were employed to enroll 25 participants for the safety comparison group.

Of the 103 candidates screened, 93 (90%) were deemed eligible to participate, and the majority of them (94%, 87/93) completed at least one real-time assessment for 3 hours when drinking.

### Procedure

After eligibility determination at study screening, the research assistant installed the mobile EMA app (MetricWire, Inc) on each participants’ personal Android or iPhone operating system smartphone. During study orientation, each participant was trained on using the app, setting alert notifications for survey prompts, and employing methods to preserve confidentiality. The purpose of the assessments was explained as assessing one’s real-time drinking behavior, subjective responses, and contextual factors (eg, location and presence of others.) for 1 or 2 drinking episodes. The participant was advised to engage in the real-time mobile monitoring during a typical drinking occasion and refrain from smoking or recreational drugs (including cannabis) during that episode. He/she was also trained on properly classifying the types of beverages consumed during mobile monitoring as “beer,” “wine,” or “liquor” using the EMA app. The TLFB calendar was used as a guide for days of the week each participant was most likely to engage in binge drinking, and he or she was encouraged to complete the EMA session(s) during those days. However, for safety and ethical reasons, the participant was not instructed or encouraged to consume a specific amount of alcohol during their future drinking episode(s). He/she was also reminded not to drive or operate machinery during or after the drinking episode and, as a safety measure, was provided financial compensation for taxi/rideshare at the end of the real-time assessment, if needed.

**Figure 1:**
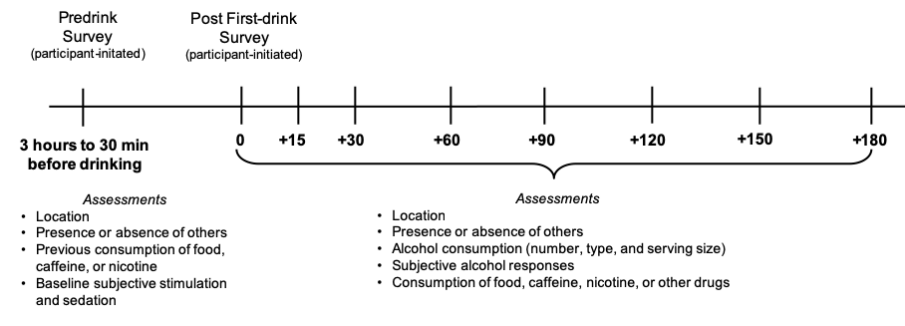
Timeline of the real-time monitoring of a drinking episode.

**Figure 2:**
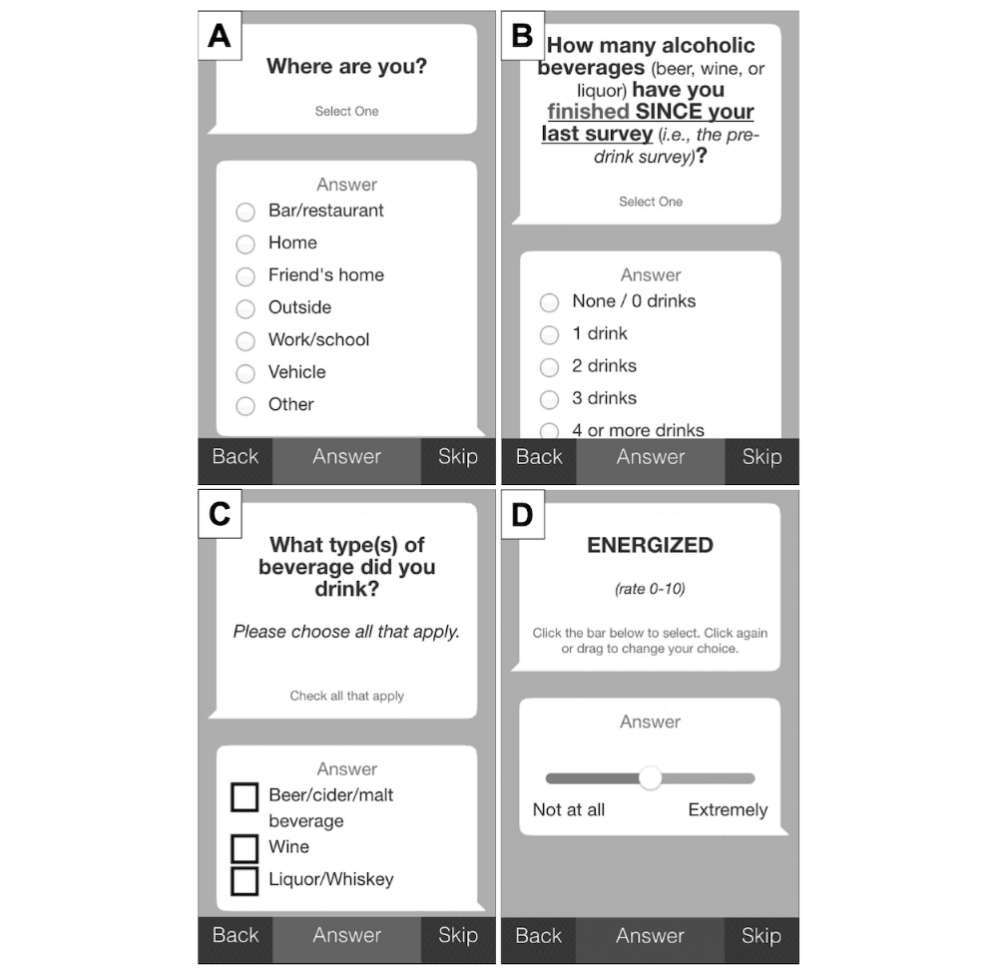
Screenshots of the smartphone interface and sample items from the real-time mobile assessments, including menu-based choices to record contextual factors (eg, (A), drinking location; (B), alcohol quantity; (C), beverage type; and (D), slider-based input to record alcohol responses).

Figure 1 depicts the timeline for the smartphone assessments. The participant was instructed to self-initiate the mobile assessment procedure by completing a predrinking baseline survey on his or her mobile device via the smartphone app 30 to 180 min before drinking alcohol. The purpose of this survey was to capture predrinking subjective responses; these outcomes will be the subject of a separate report. The participant then self-initiated the first survey after finishing their first drink. All subsequent surveys were delivered automatically to the participant’s smartphone via the EMA app at 15, 30, 60, 90, 120, 150, and 180 min thereafter. Although some previous studies have used longer EMA sessions to capture drinking behavior in young adults (eg, [14]), we opted to limit EMA session length to 3 hours in this study for 2 main reasons. First, the relatively short duration was intended to limit participant burden related to completing multiple surveys over 2 drinking episodes. Second, based on the typical pattern of alcohol consumption reported by young adult heavy drinkers in our previous studies [6,20], we expected that this assessment period would be sufficiently long to capture drinking behavior and subjective effects corresponding to ascending, peak, and early descending blood alcohol concentration (BAC) for a large proportion of participants. Of note, studies using EMA to track real-world drinking behavior and related subjective outcomes in young adults have varied in terms of the duration and frequency of monitoring [13,14] and, to date, no empirical studies have established best practice guidelines for EMA of alcohol use and subjective responses in that population [17]. The mobile surveys administered during the smartphone-based EMA sessions were designed to be brief (approximately 1 min in length) and user-friendly. As shown in Figure 2, participants used simple touch screen controls (eg, radio buttons and sliders) to enter and submit their responses to each item.

After the final survey at 180 min, the participant received a message via the smartphone app thanking them for their time and reminding them that they would be prompted to complete a next-day survey the following day. This survey was prompted automatically at 11:00 am the next day by the smartphone app. After completing the next-day survey following the first mobile drinking assessment, the participant was offered the option to complete a second mobile assessment on a separate drinking occasion for additional compensation. The second mobile assessment was identical to the first assessment and was completed at least 24 hours after the first drinking occasion. Most participants (61/83, 73%) completed a second mobile assessment, approximately 1 to 2 weeks after the first assessment (mean 11.9, SD 11.4 days). All data from the mobile assessments were uploaded wirelessly from participants’ smartphones to a secure server for analysis.

Moreover, 2 weeks after the final EMA of a drinking episode, study staff emailed the participant a link to a follow-up survey assessing drinking behavior and related consequences for the interval since completing the last real-time assessment (for details, refer to the Measures section). The participant was then compensated for his/her participation and debriefed.

### Measures

#### Smartphone Drinking Episode Assessments

The first item of the predrinking baseline survey asked whether alcohol had been consumed that day. If the response was yes, then the assessment did not proceed and the participant was reminded that the baseline survey needed to completed before drinking was initiated, and to try back again in ≥24 hours. If the response was no, that is, no alcohol consumed yet, then the 1-min baseline survey proceeded to assess context (current location; presence/absence of others; and consumption of food, nonalcoholic beverages, caffeine, nicotine, and other drugs up to the time of the survey) and baseline subjective measures.

For the post first-drink survey and 7 subsequent follow-up surveys, the participant was asked to select the alcohol type (beer, wine, or liquor), number of drinks (ie, “0,” “1,” “2,” “3,” or “4 or more”) finished since the last survey, and the size of drinks consumed. For beer, drink size options were “less than 12 oz.,” “12 oz. (bottle or can),” “16 oz. (pint/pounder),” “24 oz. (tall boy),” “32 oz.,” or “40 oz. or more”; for wine, drink size options were “5 oz. (standard glass),” “10 oz.,” or “more than 10 oz.”; and for liquor, drink size options were “1.5 oz. (1 shot),” “3 oz. (2 shots),” “4.5 oz. (3 shots),” “6 oz. (4 shots),” or “more than 6 oz*.*” Participants were also asked about current contextual variables as in the baseline survey (see prior paragraph). They also completed 10 adjective-based alcohol-subjective effects items [30,31] that will be the subject of a separate report and are not presented here.

#### Next-Day Survey

The EMA app issued a survey at 11:00 am following the conclusion of each real-world drinking assessment to capture additional information regarding that drinking episode. This *next day* survey assessed the type/brand of alcohol consumed, duration of continued drinking after the 3-hour EMA period, participants’ activities during the assessment (time spent working, socializing, standing, sitting, dancing or engaging in other physical activity, and playing games; rated from 0=“none” to 8=“a lot”), and consequences of drinking via a modified 24-item Brief Young Adult Alcohol Consequences Questionnaire (BYAACQ; [32]) assessing drinking-related consequences over the past 24 hours. Finally, an item asked the participant to indicate if the prior days’ drinking episode reflected a *typical* drinking occasion (answered “yes” or “no”).

#### Follow-Up Survey

The follow-up survey included TLFB calendar and modified BYAACQ assessing alcohol use and related consequences during the past 2 weeks, respectively, and 5 items assessing the acceptability of the real-time monitoring method. The acceptability items were as follows: (1) “Overall, the mobile app was easy to use”; (2) “The mobile assessments (surveys) were intrusive”; (3) “The mobile assessments (surveys) were too long”; (4) “I would recommend the study to other potential participants”; and (5) “Overall, I was satisfied with the study experience.” Each acceptability item was rated on a 1 to 5 scale, with 1=“strongly disagree” and 5=“strongly agree.” Participants in the safety comparison group completed an identical follow-up survey 2 weeks after their screening, excluding the acceptability items related to the smartphone assessments.

### Statistical Analyses

Feasibility was examined by (1) the percentage of nonparticipant-initiated prompts completed and (2) the estimated number of standard drinks reported at each time point. The number of estimated standard drinks consumed was used to determine the percentage of episodes that included heavy drinking. In addition, this information was used to estimate BAC (estimated BAC, eBAC) levels throughout the drinking episode according to the equation of Matthews and Miller [33]: eBAC=[(*c*/2) × (CG/*w*)]−(*β_60_* × *t*), where *c* is the number of standard drinks consumed to that point in the drinking episode, GC is a gender constant (7.5 for men and 9.0 for women), *w* is weight in pounds, *β_60_* is a constant representing the average population alcohol metabolism rate (0.017 g/dl per hour), and *t* is the time in hours since drinking began. We assumed that participants consumed the first drink in the episode over 20 min when calculating *t*, as in previous studies [10,14]. This equation approximates actual BrAC and is most accurate at BAC ≤0.08 g/dL [34].

Acceptability was determined from responses to the satisfaction items from the follow-up survey. Finally, safety of the procedure was examined by comparing drinking behavior and alcohol consequences at baseline versus the 2-week follow-up for the experimental and control group with generalized estimating equation (GEE) analyses examining group, time, and their interaction. Skewed data were log-transformed before analysis, as appropriate.

## Results

### Mobile Assessment Compliance and Study Demographics

Examination of data from the mobile assessments revealed that a high majority of participants correctly recorded their drinking during the drinking episode (83/87, 95%). Despite training participants during screening to indicate only the number of drinks finished since the prior EMA prompt, 4 participants incorrectly reported their cumulative number of drinks at each prompt instead. Thus, their data could not be interpreted, resulting in a final study sample of 83 participants. eBAC calculated for a small number of survey responses was ≥0.30 g/dl, a level of intoxication associated with loss of consciousness or death. We assumed that those time points reflected errors in reporting [14] and opted to treat reported alcohol consumption and calculated eBAC for those specific responses as missing when calculating mean number of standard drinks consumed and eBAC per time point. This resulted in dropping reported alcohol use and eBAC data from 16 survey responses (16/673, 2.4%) across all participants who completed at least one survey (n=83) and 25 responses (25/996, 2.5%) across all those who completed both surveys (n=61). Demographic and drinking information for the sample and the safety comparison group is presented in Table 1. The groups did not differ on any demographic or drinking-related variables.

**Table 1:** Demographic characteristics, baseline, next-day, and 2-week follow-up drinking and safety outcomes for the smartphone (n=83) and safety comparison (n=25) groups.

Outcome		Smartphone group	Safety comparison group	*P* value
**Demographics**				
	Age (years), mean (SD)	25.3 (2.6)	24.7 (2.5)	.26^a^
	Male, n (%)	48 (58)	15 (60)	.85^b^
	White, n (%)	54 (65)	20 (80)	.16^b^
	Education (years), mean (SD)	15.9 (1.8)	15.6 (1.9)	.42^a^
**Baseline drinking and consequences, mean (SD)**				
	% Drinking days (past month)	47.4 (18.9)	45.3 (16.7)	.61^a^
	% Binge drinking days (past month)	23.1 (10.3)	19.7 (7.7)	.13^a^
	Drinks/drinking day (past month)	4.7 (1.5)	4.1 (1.2)	.11^a^
	BYAACQ^c^ (past 2 weeks)	2.9 (2.6)	3.6 (3.0)	.46^a,d^
	Alcohol Use Disorders Identification Test total	11.1 (4.3)	11.4 (4.9)	.74^a^
**Next-day survey (smartphone group only), mean (SD)**				
	Duration of continued drinking after the 3-hour EMA^e^ period (hours)	2.7 (1.9)	—^f^	—
	Additional standard drinks consumed following the 3-hour EMA period	2.9 (1.9)	—	—
	BYAACQ (past 24 hours)	2.1 (2.1)	—	—
**Two-week follow-up drinking and consequences, mean (SD)**				
	% Drinking days (past 2 weeks)	42.8 (17.8)	40.0 (12.0)	.47^a^
	% Binge drinking days (past 2 weeks)	23.2 (11.5)	19.7 (11.7)	.19^a^
	Drinks/drinking day (past 2 weeks)	4.6 (1.6)	3.9 (1.5)	.06^a^
	BYAACQ (past 2 weeks)	4.1 (3.4)	3.4 (2.8)	.44^a,d^

^a^*t* test.

^b^Chi-square test.

^c^BYAACQ: Brief Young Adult Alcohol Consequences Questionnaire.

^d^Data log-transformed before analysis.

^e^EMA: ecological momentary assessment.

^f^Participants in the safety comparison group that did not complete the next-day survey.

### Feasibility

**Figure 3:**
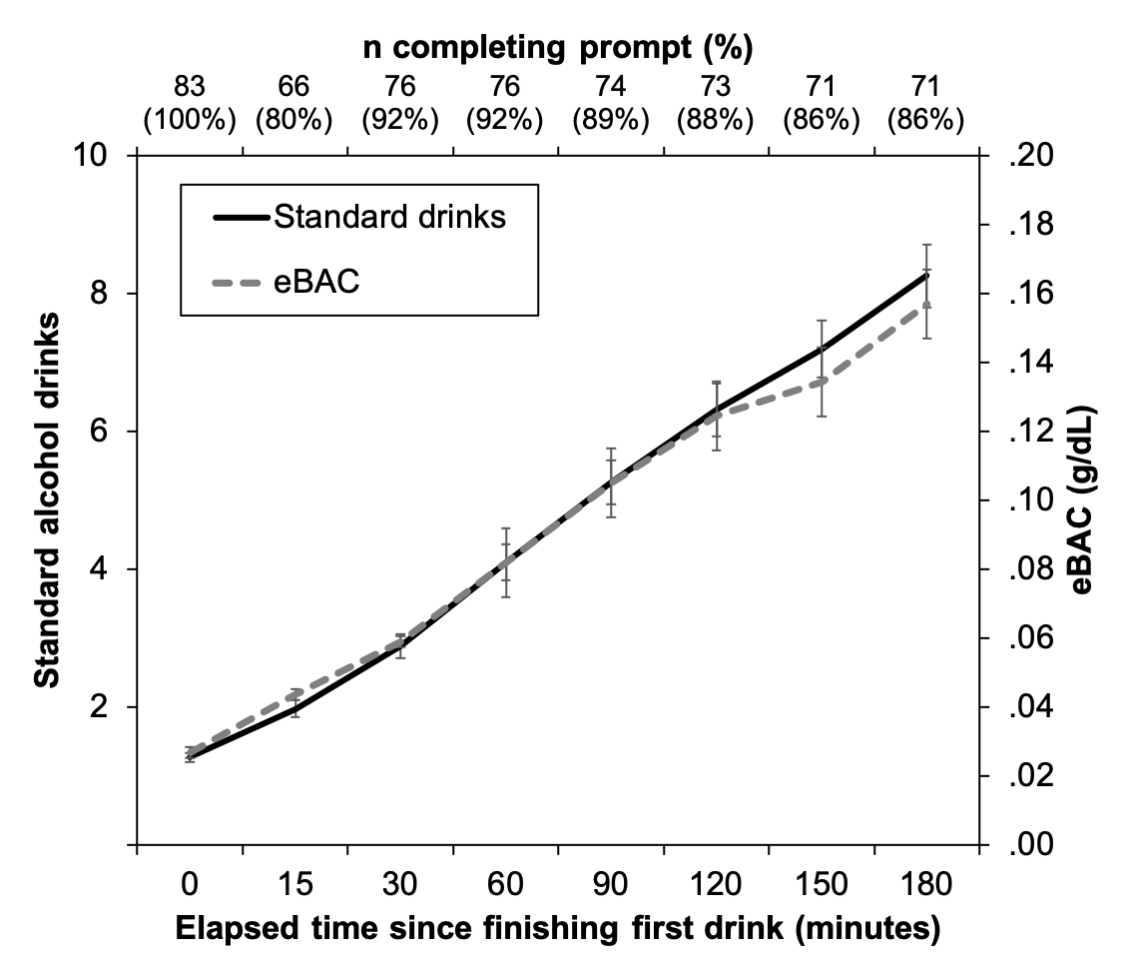
Mean (SE) of the mean standard alcoholic drinks (left y-axis) and estimated BAC (eBAC; right y-axis) at each survey time point (bottom x-axis) during the real-world drinking episode. Time 0 represents the survey that participants completed immediately after finishing their first drink in the drinking episode. The number of participants completing the survey prompt at each time point and corresponding percentage of the total study sample (n=83) is presented for each time point on the top x-axis. Drinking and eBAC data for time points where eBAC was ≥0.30 g/dl were excluded from calculations of the means for those outcomes; see the Results section for details. BAC: blood alcohol concentration; eBAC: estimated blood alcohol concentration.

All participants completed the self-initiated baseline and post first-drink survey prompts and 87.2% (507/581) of the remaining 7 system-initiated prompts over the ensuing 3 hours of the EMA session (Figure 3). Most participants (64/83, 77%) started drinking during the evening hours (ie, 5:00 pm-12:00 am) on a Thursday, Friday, or Saturday (58/83, 70%). The majority of prompts were completed in the presence of others (556/657 prompts, 84.6%) in various locations (bar/restaurant: 33.7% [221/656], one’s own home: 32.0% [221/656], or friend’s home: 24.2% [221/656]). Food consumption was not common during drinking, with participants reporting eating during only 12.2% (70/574) of prompts. Cigarette smoking or recreational drug use were also not common as they were reported during only 3.1% (18/574) and 0.2% (1/574) of prompts, respectively. For the latter, only 1 participant reported using drugs (cocaine/stimulants) during only a single prompt (0.2%).

#### Real-World Drinking Behavior

Participants reported consuming any alcohol on 75.4% (445/590) of 590 completed prompts from the self-initiated post first-drink survey onward (ie, not including the predrinking baseline survey). They consumed 7.3 (SD 3.0) standard drinks during the 3-hour drinking assessment, with eBAC reaching 0.13 (SD 0.07) g/dL at the final time point (+180 min; Figure 3). Most participants (71%, 59/83) engaged in heavy (binge) drinking during the first 2 hours of the assessment, consuming 7.4 (SD 2.8) standard drinks during that time (9.1 [SD 2.9] drinks over the entire 3-hour drinking episode), with eBAC reaching 0.14 (SD 0.06) g/dL (eBAC 0.17 [SD 0.07] g/dL at the conclusion of the EMA period). In contrast, participants who did not binge consumed 2.9 (SD 0.9) drinks during the first 2 hours of the EMA period (4.5 [SD 1.8] drinks over the entire 3-hour drinking episode), with eBAC reaching 0.04 (SD 0.02) g/dL during that time (eBAC 0.07 [SD 0.03] g/dL at the conclusion of the 3-hour monitoring period). For all participants, beer was the most common alcoholic drink consumed at each prompt (48.1%, 214/445), followed by liquor (40.2%, 179/445) and wine (16.9%, 75/445; note that percentages sum to greater than 100% because participants could report consuming multiple types of alcohol at each time point). Participants averaged 1.1 (SD 1.0) standard drinks reported at each of the 7 post baseline survey prompts.

#### Participants Completing Both Mobile Sessions

Background characteristics and alcohol consumption during drinking episodes did not differ between participants who completed 2 smartphone drinking assessments (73%, 61/83) and those who completed 1 assessment (n=22), all *P*s>.60. Among participants who completed 2 drinking episode assessments, most (>60%) had high response rates and completed all the study prompts (9/9) with an average of 8.1 and 8.3 prompts completed in the first and second episodes, respectively. There was more drinking alone during the second episode (109/504, 21.6%) than the first (78/491, 15.8%; χ^2^_1_=5.4; *P*=.02). Participants’ reported location when completing the prompts or consumption of food, nicotine, or other drugs did not differ across assessment sessions, all *P*s>.07.

### Acceptability

Participants rated the smartphone survey software as easy to use overall (mean 4.6, SD 0.7 out of 5 points) and endorsed high satisfaction with study participation (mean 4.6, SD 0.6). Similarly, participants did not consider the surveys to be time consuming (mean 2.1, SD 1.0) or intrusive (mean 2.0, SD 0.9). Overall, participants agreed that they would recommend participation in the study to others (mean 4.6, SD 0.6). Intrusiveness ratings were slightly higher among participants who completed only 1 smartphone assessment than among those who completed 2 assessments (mean 2.5, SD 1.1 vs mean 1.9, SD 0.9; *P*=.02), but other acceptability ratings did not differ based upon the number of smartphone sessions completed (*P*s>.16).

### Safety

#### Next-Day Survey

A total of 93% (77/83) of participants in the smartphone group completed the next-day survey after the real-world drinking episode. Most participants (60%, 46/77) reported continued drinking after the final survey prompt for the episode (see Table 1). Participants reported few drinking-related consequences during or after the real-world drinking episode on the modified past 24-hour BYAACQ (Table 1). The most frequently reported consequences (endorsed by ≥10% of participants) were less energy after drinking (61%, 47/77), hangover (40%, 31/77), drinking larger amounts than anticipated (14%, 11/77), and saying or doing something embarrassing as a result of drinking (13%, 10/77). None of the participants reported any serious adverse events (eg, arrest or injury) related to their drinking during the assessment period.

#### Two-Week Follow-Up Survey

GEE revealed that self-reported drinking frequency decreased at follow-up for both the smartphone and control groups (time: beta [SE]=–4.7 [1.8]; *P*=.008), but binge drinking frequency and drinks consumed per drinking day did not change for either group (*P*s>.12). There was a significant increase in self-reported alcohol consequences from baseline to 2-week follow-up (time: beta [SE]=0.4 [0.2]; *P*=.02 [data log-transformed]; see Table 1) but no main effect or interaction of group for that outcome (*P*s>.22).

## Discussion

### Principal Findings

This study demonstrated the feasibility, acceptability, and safety of using smartphone-based EMA to measure real-time alcohol use and related outcomes in young adult heavy drinkers, most of whom (71%, 59/83) completed the EMA protocol in the setting of a drinking binge. The smartphone-based approach was feasible, as evidenced by a high response rate (87.2%, 507/581) to system-initiated study prompts during the 3-hour assessment period, and acceptable, with participants reporting high satisfaction study procedures and low ratings on intrusiveness for the brief (1 min each) EMA surveys. Safety was evidenced by few drinking-related consequences and no increases in drinking quantity or frequency over the 2-week follow-up. The results of this study show that young adult heavy drinkers can complete mobile assessments of their drinking behavior and related outcomes even during very heavy drinking episodes at BAC well over the threshold for intoxication. The method described here is user-friendly and easy to administer on participants’ own smartphones and could be customized to facilitate future research on a variety of topics related to risky drinking. Furthermore, it may be less susceptible than retrospective or laboratory-based studies of binge drinking outcomes to limitations posed by recall bias and unclear ecological validity, respectively.

Relative to prior EMA studies in social drinkers, our 87% response rate to survey prompts is similar to, or higher than, response rates reported in previous studies [10,12-15]. Nearly three-quarters (71%, 59/83) of participants engaged in binge drinking during the first 2 hours and averaged just over 7 standard alcoholic drinks (eBAC 0.13g/dL) through 3 hours (see Figure 2), which is considerably higher than the 2 to 4 drinks reported in prior EMA studies [10,11,14,35]. In contrast to those studies, however, this study recruited heavy social drinkers specifically (ie, light drinkers and those with severe AUD were excluded from participation) and limited measurement of alcohol use and outcomes for up to 2 self-selected drinking events. Previous EMA studies have recorded drinking behavior and outcomes over longer durations (eg, 3 weeks [14]), which can provide data on heavier- and lighter-drinking events. Of note, the drinking episode was not an isolated event for the study participants, with the vast majority (85%, 66/77 completing the next day survey) reporting that it was indicative of their *typical* drinking.

Regarding feasibility and acceptability of the EMA approach, participants reported good compliance with study directions to avoid alcohol or other drug use during the assessments and high overall satisfaction with study procedures. The data from this study suggest that heavy drinkers can comply with instructions to avoid those substances even during binge episodes with high BAC and that this approach can produce data on drinking-related outcomes that are contaminated only minimally by other substance use. The high acceptability ratings reported in this study echo previous findings from EMA studies in general young adult drinker samples [13,36]. The brief duration of the mobile surveys (approximately 1 min each) may have facilitated the overall high acceptability of the mobile procedure despite the repeated survey prompts throughout the 3-hour drinking episode. Furthermore, the high rates of smartphone use among young adults and participants’ high degree of familiarity with these devices may have contributed to the high overall satisfaction and usability ratings reported in this study.

Importantly, the results of this study suggest that EMA of a real-world binge drinking episode in heavy drinkers is safe. Participants reported few alcohol-related problems during the drinking episode, and those that were reported tended to be minor (eg, hangover and less energy after drinking). Both groups reported a small decrease in their frequency of alcohol use from baseline to 2-week follow-up, with no changes in binge drinking frequency. This finding is in line with the results of previous studies indicating that EMA of alcohol use is associated with minimal or no changes in reported drinking behavior [11,37-39]. However, studies of reactivity to multiple assessments of alcohol use via retrospective self-report or interview (eg, TLFB) spaced several weeks/months apart have shown that, in general, self-reported alcohol use and consequences decrease over time with those repeated assessments (for a review, refer to the study by Schrimsher and Filtz [40]). In contrast, we observed a small, but significant, increase in self-reported alcohol consequences over the 2-week follow-up period across all participants, but all reported consequences tended to be mild, with no serious adverse events reported. This effect appeared to be driven mainly by the small (approximately 1 point) increase in mean BYAACQ scores from baseline to 2-week follow-up among the smartphone group (Table 1), although our GEE model did not show a significant effect or interaction of group for that outcome. We speculate that completing the next-day assessments of alcohol-related consequences related to the real-world drinking episodes increased awareness of those consequences among the smartphone group, resulting in them reporting slightly higher scores on the 2-week follow-up BYAACQ relative to baseline. However, this study was not designed to test this hypothesis directly, and to our knowledge, no previous studies have examined the effect of monitoring drinking on perceptions of alcohol-related consequences over time. Whether EMA of alcohol use affects individuals’ awareness of drinking-related risks may be a promising area for future research.

### Strengths and Limitations

This study featured several strengths, including a well-characterized sample of young adult heavy drinkers, application of existing mobile phone technology to support EMA of real-world heavy drinking and related outcomes, and inclusion of an independent heavy drinking sample as a safety comparison group. However, there are also some limitations worth noting. First, although we recruited current binge drinkers, light drinkers and those meeting criteria for severe AUD were excluded from participation. Thus, based on the current data, we cannot infer the feasibility, acceptability, or safety of our smartphone-based EMA approach in those groups. Second, the duration of our monitoring period (3 hours per drinking occasion, over a maximum of 2 occasions) was brief and fixed, in contrast to previous studies that have assessed alcohol use outcomes over several weeks [11] and scaled the duration of the EMA sessions according to participant-reported drinking patterns [14]. Participants in this study reported continued drinking for nearly 3 hours after the EMA period ended, consuming approximately 3 additional drinks during that period. Additional research is needed to evaluate the feasibility, acceptability, and safety of using this assessment method over longer drinking episodes, including any potential effect of a longer monitoring duration on compliance with the survey prompts. Third, the protocol used in this study relied upon participant self-report to estimate alcohol consumption and BAC, and the equation we used to compute eBAC is less accurate at BAC ≥0.08 g/dL [34]. Future research on EMA of drinking behavior may benefit from incorporating recently developed wearable alcohol biosensors, which can measure consumption passively at the skin, to provide an objective measure of alcohol use [41]. Fourth, for the purposes of this study, “safety” was conceptualized in terms of scores on the next-day BYAACQ (for the smartphone group) and changes in drinking behavior or self-reported alcohol-related consequences on the BYAACQ at 2-week follow-up, compared across the smartphone and safety comparison groups. We did not examine other safety-related outcomes, such as the potential for the smartphone prompts to serve as a distraction while walking or driving (although we note that participants were instructed not to drive after consuming alcohol during the study), nor did we collect next-day report data from members of the safety comparison group, which prevented us from examining possible group-related differences on consequences related to real-world drinking events. Future studies could implement a broader definition of “safety” to evaluate the potential effects of EMA of alcohol use on a wider range of safety-related outcomes. Finally, this study does not speak to the reliability and validity of using smartphones to measure alcohol use and related outcomes during real-world (binge) drinking events. We will present data on the reliability of the smartphone-based EMA method across multiple real-world drinking sessions and validity of the approach relative to the laboratory alcohol challenge session in a separate report.

### Conclusions

In sum, the results of this study provide preliminary support for the feasibility, acceptability, and safety of using smartphone-based EMA to assess alcohol use and related outcomes (eg, subjective responses and drinking context) in young adult heavy drinkers. These data suggest that young adults can use smartphones to monitor their drinking even during very heavy drinking episodes, with average consumption approximately double (or more) that of previous work in this field [10,11,14,35]. Future research could use this technology to further study the dynamics of binge drinking behavior, refine EMA-based treatment approaches targeting risky drinking specifically [42,43], or develop “just-in-time” interventions to reduce heavy drinking and associated risks in young adults [44]. In sum, smartphone-based EMA has great potential to provide a practical, inexpensive, and efficient way to capture a large amount of data on real-world drinking behavior and associated consequences, which may inform future research on the epidemiology of and intervention for AUD.

## Abbreviations

AUD: alcohol use disorder
BAC: blood alcohol concentration
BrAC: breath alcohol concentration
BYAACQ: Brief Young Adult Alcohol Consequences Questionnaire
DSM-5: Diagnostic and Statistical Manual of Mental Disorders, Fifth Edition
eBAC: estimated blood alcohol concentration
EMA: ecological momentary assessment
GEE: generalized estimating equation
TLFB: Timeline Followback
